# Supplementary material for: Are carbon emissions trading and green financial instruments synergistic? -Comprehensive quantitative research based on content analysis
Source: PLoS One. 2024 Mar 7;19(3):e0298601. doi: 10.1371/journal.pone.0298601 (PMC10919601; doi:10.1371/journal.pone.0298601)
Supplement: S1 Data — (ZIP) [file pone.0298601.s001.zip › Data/地级市Z_F工作报告.zip!/╕╩╦α/╞╜┴╣╩╨/└┤╫╘╣1⁄2╓┌║┼í╛┬φ┐╦╩2╛▌═°í┐_2016.docx]

各位代表：

现在，我代表市人民政府，向大会作工作报告，请予审议，并请政协委员和列席人员提出意见。

“十二五”工作回顾

“十二五”以来，面对复杂多变的宏观经济形势和艰巨繁重的改革发展任务，在市委的坚强领导下，在市人大、市政协的监督支持下，我们坚持以党的十八大和十八届三中、四中、五中全会精神为指导，深入贯彻习近平总书记系列重要讲话精神，紧紧依靠全市广大干部群众，紧盯全面脱贫和全面小康战略任务，始终扭住市三次党代会确立的“加快转型升级，促进科学发展，建设小康和谐文明生态平凉”的思路目标，突出转变经济发展方式这一主线，牢牢把握稳中求进总基调，主动适应经济发展新常态，全力稳增长、调结构、促改革、惠民生，全市经济持续较快发展，人民生活水平显著提升，社会大局和谐稳定，谱写了平凉发展浓墨重彩的崭新篇章。

五年来，我们坚持抓项目、促消费，全力以赴稳增长，经济实力迈上新的台阶。全力实施以“十大工程”为重点的“3341”项目工程，五年共实施500万元以上项目4663项，完成固定资产投资2289.9亿元，年均增长17.7%，是“十一五”时期的3倍。认真落实促进消费各项政策措施，积极培育新的消费热点，陇东农副产品批发交易中心、宏达国盛商贸建材市场、金果博览城等一批商贸物流设施建成投用，城乡消费日趋活跃，家政服务、健康养老、电子商务等新型业态不断涌现，全市社会消费品零售总额预计达到176.5亿元，年均增长14.9%，五年翻了一番。2015年全市地区生产总值预计达到356亿元，年均增长10.5%；大口径财政收入48.1亿元、公共财政预算收入24.7亿元，分别是“十一五”末的1.6倍和1.8倍，财政支出突破160亿元，年均增长13.5%。

五年来，我们坚持抓转型、促升级，持之以恒调结构，产业质量效益显著提升。突出提质增效、延伸链条，改造提升传统产业，加快培育战略性新兴产业，三次产业结构由“十一五”末的21.8:46.9:31.3调整到26.1:29.8:44.1。工业转型升级迈出坚实步伐，新增探明煤炭储量23亿吨，《陇东能源基地开发规划》、《灵台矿区总体规划》获批，五举、赤城、邵寨煤矿等矿井项目和红河油田百万吨产能建设稳步推进，崇信电厂一期并网发电，全省首个煤制甲醇项目建成投产，国家级科技示范项目20万吨聚丙烯生产线启动实施，煤电化一体化开发取得重大进展，煤炭就地转化率提高14.5个百分点；天纤棉业、兴旺管材、宝马木浆纸、欣叶纸箱精包装、华星专用车改装、庆华陶瓷等非煤产业项目相继建设，光伏发电、碳纤维复合材料、生物胶原蛋白肽等新兴产业项目建成投产，非煤工业占规上工业增加值比重提高10.7个百分点。持续实施现代农业“五个百万”增收工程，大力推广全膜双垄沟播等旱作农业技术，肉牛饲养量居全省农区之首，苹果产业成为全市覆盖面最广、产业链价值链带动力最强、带农增收效果最为显著的支柱产业，我市成为黄土高原苹果优势种植区的核心区域，“平凉金果”打入欧盟高端市场，设施蔬菜规模不断扩大，粮食总产连续5年保持在百万吨以上，引进建办了秦宝牧业、海升果业、正大饲料、方盛蔬菜等一批农业产业化龙头企业，农民专业合作社、家庭农场等新型农业经营主体进一步壮大，特色产业在农村居民可支配收入中的比重达到51%，全市农业增加值预计比“十一五”末增长36%以上。集中实施华夏文明传承创新区“163”计划，崆峒山、大云寺·王母宫大景区建设加快推进，实施原生态民俗文化体验区、崆峒生态旅游度假山庄等重点文化旅游项目103个，新增文化企业593家，“神奇秀美崆峒山、天下养生第一地”的知名度和影响力不断扩大，旅游接待人数和综合收入分别达到1300万人次和70.5亿元，比2010年分别增长2.1倍和2.4倍，文化产业增加值年均增长24%。

五年来，我们坚持抓双联、补短板，聚力攻坚促脱贫，扶贫开发取得重大成就。扎实推进“1236”扶贫攻坚和双联行动，动员全社会力量积极参与扶贫帮困，省、市、县936个单位联系1117个村，3万多名干部联系4.5万贫困户，落实帮扶项目6322个，帮办各类实事6.9万件。突出改善路水电房等基础条件，五年共实施整乡整村推进项目303个，完成农村危旧房改造11.3万户，易地扶贫搬迁1.08万户，彻底告别了土窑洞和地坑庄子，新建改建农村公路7029公里，建制村沥青（水泥）路通畅率达到94%，解决了56.6万人饮水安全问题，初步实现了“自来水化市”和“梯田化市”目标。加大适用技术培训力度，大力发展牛、果、菜和劳务输转等富民多元产业，贫困群众自我“造血”功能显著增强，收入水平较快提升。全市贫困人口由2011年的80.3万人减少到2015年的31.95万人，贫困面由41.54%下降到16.43%。

五年来，我们坚持抓基础、建平台，持续不断强后劲，城乡面貌发生了巨大变化。西平、天平铁路、西长凤高速和静庄、泾渗、韩张等二级公路建成通车，等级公路网密度比“十一五”末提高一倍多，联结城乡、贯通内外的路网体系基本形成。引洮二期静宁受水区和抗旱水库等重大水源骨干工程开工建设，新建改扩建330千伏、110千伏输变电工程12处，水利、电网保障能力显著增强。光纤宽带传输网络辐射城乡，有线宽带和3G、4G信号实现乡镇全覆盖。坚持规划引领、中心带动，统筹推进新型城镇化，五年累计完成城乡建设投资450亿元，是“十一五”时期的3倍，城镇化率达到36.5%。着力打造“一中心两园区”城市布局，相继实施了东大门改造、西大门畅通、绿地公园、八沟一河治理、棚户区改造、地下管网等一批节点改造、功能配套、景观提升工程，构建了“六横十二纵”路网框架。建成了中心城区热电联产集中供热工程和西北首个水泥窑协同处理城市生活垃圾示范项目，实现了主城区建成道路雨污管网全覆盖、垃圾无害化全处理，集中供热面积达到1100万平方米。建成区面积达到42平方公里，绿地率提高到31.8%，城市功能和品位显著提升。六县县城和小城镇建设取得明显成效，抓建城乡一体化试点乡镇24个，建成美丽乡村、“三清五改”和新农村建设示范村1318个，撤乡改镇15个。把园区作为聚集要素、支撑发展的重要平台，平凉工业园区管理体制进一步理顺，崆峒山生态文化旅游示范区开发加快推进，工业园区（集中区）、农业示范园、文化产业园区的承载能力明显增强。加强生态建设和环境保护，治理水土流失1176平方公里，新修梯田118.7万亩，完成人工造林115万亩，森林覆盖率达到30.9%，主要污染物排放和节能降耗指标全面完成，环境质量明显改善，平凉成为全省唯一的“国家级生态示范区”。

五年来，我们坚持抓开放、重创新，突出重点促改革，发展活力明显增强。进一步转变政府职能，大力推进简政放权，市级行政审批事项减少71%，非行政许可事项全面取消。建成了市县两级“三张清单一张网”，并联审批、网上审批积极推行，行政审批进一步规范。商事制度改革全面实现了“三证合一”和“一照一码”登记，市场主体达到8.8万户，是“十一五”末的2.1倍。深化财税金融改革，“营改增”范围不断扩大，国库集中支付和公务卡应用改革全面推行，财政存量资金有效盘活，化解政府债务11.9亿元。设立了市长金融奖，组建了惠民、泓源、富民等投融资企业，成功发行企业债券15亿元，实现了市场化融资的重大突破。甘肃银行平凉分行、交通银行平凉分行开业运营，新增便民金融服务网点2354个。国资国企改革、农村综合改革积极推进，水利改革试点全面完成，农村集体土地三权确权登记发证、市级政策性农业保险全面推开，培育新型农业经营主体2618个。开放合作不断深化，成功举办了第九届中国民间艺术节、崆峒文化旅游节、平凉金果博览会、第六届世界养生大会、皇甫谧世界针灸文化交流会、华夏母亲节暨海峡两岸西王母女性文化研讨会等重大节会，引进全国500强企业12户，江苏悦达、陕西星王、苏宁云商、中电科、甘肃陇能、酒钢集团等一批大型知名企业落户平凉。大力推动科技创新，新增省级工程技术研究中心2个，研发新产品45项，取得省级新产品及科技成果26项，科技对经济发展的贡献率明显提升。

五年来，我们坚持抓保障、促和谐，尽心竭力惠民生，人民生活水平大幅提升。把增进民生福祉作为一切工作的出发点和落脚点，每年承诺措办一批惠民实事，着力解决群众行路、就业、上学、就医、住房等突出问题，财政用于民生的支出累计达到498.4亿元，是“十一五”时期的2.6倍，占财政总支出的80%。全面落实促进创业就业各项政策，高校毕业生平均就业率保持在88%以上，五年累计新增城镇就业18.4万人。持续推进社保扩面提标，全市新农合补助、城市低保、农村低保、五保户供养标准分别比2010年提高200%、86%、186%和136%，最低工资标准从670元提高到1370元，失业保险月平均标准由370元提高到936元。临时救助制度全面建立，城乡居民大病医疗保险启动实施。建成保障性安居工程4万多套，是“十一五”时期的4倍。被征地农民养老保险稳步推进，乡镇干部基本工作生活条件“三年改善工程”取得显著成效。城乡居民人均可支配收入预计分别达到20995元和6060元，比“十一五”末增长78.4%和93.2%。新建改建校舍265.2万平方米，乡镇中心幼儿园实现了全覆盖，全市顺利通过“两基”国检，华亭、崇信、泾川、灵台4县通过国家县域义务教育均衡达标验收，平凉职业技术学院一期工程建成，平凉医专升格为甘肃医学院，填补了全省省属西医本科院校和我市本科院校的空白，全市人均受教育年限由2010年的8年提高到2015年9.8年。建成市妇幼保健院、全科医师培养基地等市县级医疗机构36个，改扩建乡镇卫生院79个，新建标准化村卫生室681个，每千人拥有床位数五年实现了翻番。全面推进中医药工作和健康促进模式改革，患者次均住院费用为全省最低。市广电新闻中心建成运行，县区数字影院、农家书屋实现了全覆盖，全民体育健身活动蓬勃开展。

五年来，我们坚持抓服务、转职能，从严从实改作风，政府自身建设不断加强。突出为民务实清廉，加快建设法治政府、廉洁政府和服务型政府，市级政府新一轮机构改革全面完成，调整理顺了食药监、工商、质监、城市执法等管理体制，事业单位分类改革稳妥推进。市公共资源交易中心成立运行，政务服务中心实现市县乡三级全覆盖，重大决策、公共资源配置、项目审批、财政预决算、“三公”经费等信息全面公开。加快推进依法行政，组建了市政府法律专家咨询委员会，健全了重大行政决策公众参与、专家论证、风险评估、合法性审查和集体讨论决定制度。严格落实行政执法责任制，细化行政处罚自由裁量权标准，行政执法行为进一步规范。自觉接受人大法律监督、政协民主监督和社会舆论监督，建立向人大报告和向政协通报工作常态化机制，积极配合人大、政协开展视察调研，五年共办理人大代表建议405件、政协委员提案524件。扎实开展党的群众路线教育实践活动和“三严三实”专题教育，严格落实中央八项规定和省委“双十条”、市委“十二条”规定，大力精减会议文件，改进督查方式，加强调查研究，规范公务活动，清理公务用车和办公用房，全市“三公”经费支出逐年压减。认真落实党风廉政建设“3783”主体责任，健全完善责任分解、压力传导、履责报告等机制，加强对公务人员特别是领导干部的经常性教育和监督管理，强化审计监督和行政监察，严肃查处发生在群众身边的“四风”和腐败问题，深入开展纠风治乱，政府系统廉政建设不断加强。深化普法教育，创新社会治理，平安建设深入推进，一些影响社会稳定的突出矛盾和信访积案得到有效化解。食品药品安全监管水平进一步提升，安全生产形势持续稳定。全面贯彻落实党的民族政策和宗教工作方针， “两个共同”示范区建设扎实推进。深入开展双拥共建活动，我市连续两次获得“全国双拥模范城”称号。统计调查、档案、外事、侨务、地震、气象、人防、地方志、红十字、残疾人、老龄等事业取得新的成效。

刚刚过去的2015年，全市上下认真落实市委三届九次全委扩大会暨全市经济工作会议精神，围绕市三届人大五次会议确定的目标任务，积极应对经济下行压力，认真履职，攻坚克难，全市呈现出经济平稳增长、改革有序推进、民生持续改善、社会和谐稳定的良好局面。精准扶贫精准脱贫工作扎实推进。制定了 “2+19”一揽子精准扶贫精准脱贫政策措施，建立了覆盖县、乡、村、户、人的五级扶贫信息管理系统，整合各类财政支农专项资金12.6亿元，发放双联惠农、精准扶贫专项贷款22亿元，实施产业培育、基础改善、公共服务项目1195个，改造县乡道路119公里，建成通村硬化路1367.8公里，解决了3.7万人的饮水安全问题，改造农村危旧房3.37万户。输转劳动力48万人，创劳务收入80.6亿元。全市预计完成减贫8.65万人。现代农业“五个百万”增收工程深入实施。泾川县被认定为省级现代农业示范区，创建国家和省级苹果标准化示范园9个，建成标准化肉牛养殖小区33个、蔬菜示范基地15个，认证“三品一标”农产品生产基地23.2万亩，粮食总产实现“十连增”，肉牛饲养量、出栏量分别达到120.7万头和44万头，果品、蔬菜总产量分别达到120万吨和143万吨，推广种植油用牡丹2.7万亩，培育了鑫海食品、银海生物复混肥等一批成长性强的龙头企业。全力稳定工业经济发展。持续推进陇东能源化工基地建设，唐家河煤矿取得国家发改委“路条”，华泓汇金煤化工、陇能煤转化等项目前期工作取得较大进展。新上了农业光伏发电、新型建材、矿山机械再制造等一批新能源、新材料和循环经济项目。认真落实“双创”各项政策措施，优化发展环境，新增中小微企业1512户。现代服务业发展势头强劲。中华崆峒养生地建设开局良好，崆峒山、大云寺·王母宫大景区总体规划编制完成，泾川花样年美年文化城开工建设。平凉工业园区综合物流中心全面建成，义乌国际商贸城、泓泰商贸城等一批商贸物流设施和专业市场加快建设，淘宝网“特色中国·平凉馆”建成运营，新兴服务业发展迅速。预计全市三产增加值增长10.5%。基础设施建设力度加大。平天、彭大高速、平华一级公路、六盘山至平凉750千伏输变电工程，以及庄浪花崖河、泾川朱家涧、崇信关河、崆峒北杨涧4座抗旱水库等重大项目开工建设。全市固定资产投资预计达到602亿元，增长12%。招商引资成效明显。坚持精准招商、定向招商、以商招商，先后与中冶赫山、瑞合能源、山东大海、广州力柏、大连软件园等企业集团进行对接洽谈，30万吨二甲醚、华信百万亩油用牡丹、微风发电、万达广场、红星美凯龙生活广场等一批投资过10亿元的项目正式签约，落实到位资金519.8亿元，增长22.5%。新型城镇化稳步推进。新版《平凉市城市总体规划》经省政府批复实施，省列新型城镇化试点县镇“多规合一”规划编制稳步推进。中心城市“十大项目、六大突破”工程建设进展顺利，新民中路、柳湖路西段、北后街道路改造和东湖公园节点改造提升工程全面完成。抢抓政策机遇，片区化、规模化推进棚户区改造，全市完成房屋征收2148户49.3万平方米，改造棚户区6725户。保障和改善民生更加有力。承诺措办的10件21项实事全面完成。新建改扩建校舍10万平方米，建成标准化学校428所，新建标准化村卫生室30个，建成村级体育健身工程100个、乡村舞台425个，市老年人活动中心、市博物馆开工建设，平凉体育健身广场、市残疾人康复中心建成投用。甘肃医学院、平凉农校、平凉师范部分体育运动区定期面向社会开放。“十三五”规划编制扎实有效。认真贯彻中央和省、市委“十三五”规划《建议》，深入调研论证，广泛征求意见，精心谋划对接，全市“十三五”《规划纲要》及44个专项规划草案全部完成，正在按程序审订。

各位代表，过去的五年，是我市经济加速发展、综合实力提升最快的五年，是转变发展方式、调整结构步伐最快的五年，是加大扶贫攻坚、实现脱贫人口最多的五年，是突破基础瓶颈制约、城乡面貌变化最大的五年，是创新投融资体制、金融支持发展力度最大的五年，是社会和谐稳定、人民群众得到实惠最多的五年。在经济下行压力加大、资源环境约束趋紧、发展基础薄弱的情况下，我们能够取得这些巨大成就，是省委、省政府和市委坚强领导的结果，是市人大、市政协监督支持的结果，是全市广大干部群众艰苦奋斗的结果。在此，我代表市人民政府，向全市各族人民，向各民主党派、工商联、无党派人士和人民团体，向中省驻平单位，向驻平部队和武警官兵，向所有关心支持平凉发展的各界人士，致以崇高的敬意和衷心的感谢！

各位代表，五年的成就令人振奋，积累的经验弥足珍贵。我们深深体会到，要把全市人民的事情办好，实现全面脱贫、全面小康的宏伟目标，必须始终坚持发展第一要务，突出大项目带动、大招商助推、大产业支撑、大园区引领，持续做大经济总量，不断优化经济结构，着力提高质量效益，推动全市发展行稳致远；必须始终坚持改革创新，注重处理好政府与市场的关系，着力破解制约科学发展的体制机制障碍，以改革激发活力，以创新引领发展；必须始终坚持依法行政，强化法治理念，规范行政行为，推进简政放权，用法治思维和法治方式深化改革、推动发展、化解矛盾、维护稳定；必须始终坚持以人为本，充分调动人民群众的积极性、主动性和创造性，着力解决群众最关心、最直接、最现实的利益问题，真正做到发展为了人民、发展依靠人民、发展成果由人民共享；必须始终坚持改进作风，切实践行“三严三实”要求，强化责任担当，紧盯认准的目标不动摇，一张蓝图绘到底，一届接着一届干，全力以赴抓落实、干到位、求实效。

各位代表，在总结成绩经验的同时，我们也清醒地认识到，受宏观经济下行、煤电市场低迷、投资动能不足等因素影响，全市经济增速回落，地区生产总值、规模以上工业增加值等经济发展指标没有达到“十二五”预期。全市经济社会发展和政府工作中还存在不少差距和问题：一是经济总量小、人均水平低，产业结构不够合理，支撑发展的大项目尤其是工业项目少，科技创新能力不足，新的经济增长点培育缓慢，调整结构、转型升级的任务艰巨。二是交通、水利、信息等基础设施瓶颈制约还没有完全破解，人才、资金短缺的问题依然突出，资源环境约束日益加剧。三是教育、文化、医疗等社会事业还不能满足人民群众的现实需求，基本公共服务水平有待进一步提升。四是城乡居民收入水平还不高，就业渠道不宽，尤其是农业应对自然灾害和市场风险的能力弱，农民持续增收的难度加大，部分群众生活还比较困难，精准扶贫精准脱贫的任务繁重。五是政府职能转变还不到位，少数干部担当精神、责任意识不强，行政效率和服务质量不高，个别部门单位还存在懒政怠政不作为的现象。对于这些问题，我们将采取有力措施，认真加以解决。

“十三五”思路目标和战略重点

“十三五”时期，是我市全面建成小康社会的决胜阶段，是深化改革扩大开放的攻坚阶段，也是经济社会发展转型升级的关键阶段。虽然我们面临着发展大环境不确定因素增多、经济下行压力持续加大、区域竞争更趋激烈等严峻挑战，但全市发展总体仍处于可以大有作为的重要战略机遇期。国家深入实施西部大开发、“一带一路”建设、六盘山片区扶贫开发、陕甘宁革命老区振兴等重大战略规划，大力推进扶贫攻坚工程，为我们改善基础条件、完善公共服务、加快脱贫步伐、扩大开放开发提供了更多政策支持；创新驱动战略的深入实施、东部产业的梯度转移，为我们加快转变发展方式、推动产业转型升级增添了新的动力；特别是经过多年的快速发展，我市产业、区位等后发优势日益凸显，内生动力和发展活力不断增强，广大干部群众盼小康、快发展的愿望强烈，为 “十三五”发展打下了坚实基础。只要我们始终保持奋发有为的精神状态，积极有效地应对各种风险和挑战，科学谋划，真抓实干，趋利避害，主动作为，就一定能够如期实现脱贫目标、全面建成小康社会。

“十三五”全市经济社会发展的总体要求是：高举中国特色社会主义伟大旗帜，全面贯彻党的十八大和十八届三中、四中、五中全会精神，以马克思列宁主义、毛泽东思想、邓小平理论、“三个代表”重要思想、科学发展观为指导，深入贯彻习近平总书记系列重要讲话精神，牢牢扭住全面建成小康社会、全面深化改革、全面依法治国、全面从严治党的战略布局，切实贯彻创新、协调、绿色、开放、共享的发展理念，全面落实省委坚持中高增速与质量效益并重等“八个并重”的要求，积极适应新常态，始终坚持发展第一要务，全力打赢脱贫攻坚战，持续深化改革，破解瓶颈制约，保障改善民生，增进人民福祉，统筹推进经济、政治、文化、社会和生态文明建设，加快建设小康和谐文明生态平凉，确保到2020年与全国全省一道全面建成小康社会。

统筹考虑“十三五”发展环境、发展基础和增长潜力，今后五年的主要目标是：

——综合实力跨上新台阶。经济保持中高速增长，主要经济指标平衡协调，到2020年全市地区生产总值跃上500亿元台阶，年均增长8％左右，高于全省平均水平，比2010年翻一番；人均生产总值达到24000元，年均增长8％左右；公共财政预算收入达到30亿元以上，年均增长8％以上。

——转型升级取得新成效。经济结构趋于合理，发展质量效益明显提高，创新能力显著增强，文化旅游、农产品加工、煤电化冶、商贸物流四大产业集群日益壮大，产业高端化、集群化进程不断加快，工业化与信息化融合程度不断加深，农业现代化水平明显提升，现代服务业发展步伐加快，新产业新业态不断成长，新兴产业、服务业增加值占生产总值比重分别达到15%和48%以上，非公经济比重达到60%以上。

——基础设施建设取得新突破。铁路、公路、航空运输网络更加完善，实现市通高铁航运、县县通高速、乡乡通国省道、村村通沥青（水泥）路，水利保障和防灾减灾水平大幅提高，农村自来水实现全覆盖，信息基础设施建设和开发应用进一步加强。城乡面貌持续改善，承载功能大幅提升，城镇布局更加优化，新型城镇化加快推进，城乡一体化水平显著提高，具备条件的村庄基本建成美丽乡村，常住人口城镇化率达到50％以上。

——生态环境质量得到新改善。生产方式和生活方式绿色、低碳水平明显上升，污染治理和生态修复实现突破，单位生产总值能耗、主要污染物排放总量和单位生产总值二氧化碳排放量控制在省上下达目标之内，空气和水环境质量保持良好，能源资源开发利用效率大幅提高，政府、企业、公众共治环境治理体系基本形成，生态文明制度体系基本建立，建设天蓝地绿水清、宜居宜业宜游的绿色生态平凉。

——改革开放开创新局面。重点领域和关键环节改革取得实质性突破，各方面制度机制更加成熟更加定型，改革红利充分释放。开放型经济加快发展，招商引资水平显著提高，进出口贸易规模效益不断提升，2020年进出口总额达到3.8亿元以上，年均增长10％以上。

——社会文明达到新水平。中华民族伟大复兴的中国梦和社会主义核心价值观更加深入人心，公民思想道德素质、科学文化素质、法治素质明显提高。公共文化服务体系基本建成，文化旅游产业成为支柱产业，文化影响力进一步扩大。民主法治更加健全，社会治理体系更加完善，社会更加和谐稳定。

——人民生活水平实现新提升。城乡居民收入比2010年翻一番，现行标准下农村贫困人口实现脱贫，贫困县全部摘帽，解决区域性整体贫困。就业更加充分，教育、文化、医疗、住房等公共服务体系更加健全，社会保障体系覆盖城乡居民，基本公共服务均等化水平稳步提高，人民群众生活质量、健康状况、居住环境显著改善。

重点实施六大战略任务：

（一）聚焦全面脱贫，突出农民增收和补齐短板，全力打赢脱贫攻坚战。

把扶贫开发作为建成全面小康社会的最大任务，坚持扶贫攻坚与双联行动深度融合，落实“853”精准脱贫管理办法，建立贫困户收入状况精准监测和脱贫认定机制，分类扶持，动态管理。深入实施“五个一批”工程，大力培育富民多元产业，支持发展劳务经济，加快实施易地搬迁，推进生态补偿脱贫和教育扶贫、健康扶贫工程，落实政策性兜底保障，提高扶贫成效。加快贫困乡村基础设施建设，推进基本公共服务均等化，实现贫困村硬化路、自来水、动力电、宽带信息网络、安全稳固住房和村幼儿园、标准化村卫生室、综合公共文化服务中心等全覆盖。创新扶贫投入方式，建设县级扶贫开发资金项目整合管理平台，加大金融扶贫力度，实现贫困村农民资金互助社全覆盖。探索资产收益扶贫方式，引导贫困户利用土地、房产、林地等资产入股龙头企业、合作社等经营主体，获得分红收益，拓宽增收渠道。强化政府引导，动员社会力量参与，形成精准扶贫精准脱贫的强大合力。

（二）突出创新驱动，加快构建现代产业体系，全力打造全省经济转型升级先行区。

突出科技创新在全面创新中的引领作用，强化企业创新主体地位，以创新推动产业转型升级，加快构建结构优化、技术先进、清洁低碳、附加值高的现代产业体系。加快转变农业发展方式，健全现代农业产业体系、生产体系、经营体系，大力推广旱作农业技术，稳定粮食生产，持续提高牛、果、菜优势产业的质量效益和竞争力，培育设施养殖、马铃薯、油用牡丹、中药材等多元增收产业，推动农业标准化、信息化和机械化，因地制宜发展家庭农场、农民合作社，培育壮大农业企业，创建不同类型、不同层级的现代农业示范园，建成全国牛果菜生态循环农业示范区，建设百万亩木本油料生产基地。坚持实施工业强市战略，围绕煤电化冶一体化发展，着眼绿色开发、深度转化、高效利用，全力实施《陇东能源基地开发规划》和《灵台矿区总体规划》，争取实施陇东特高压电力外送工程和一批火电项目，大力发展甲醇、聚丙烯、烯烃、煤制气等煤化工产业，推进华亭、灵台两个现代化安全高效矿区建设，打造平凉工业园区、华亭工业园区两个千亿级煤电化冶循环经济示范基地和千万千瓦级大型煤电基地。以延伸链条和产业集聚为核心，加快装备制造、建材、农产品加工、棉纺和纸制品生产等传统产业改造升级，建成高端装备制造产业园、区域建材基地、西北纸制品包装基地和重要的棉纺产业基地。加快发展战略性新兴产业，培育一批具有区域影响力的创新型企业，建成光伏、碳纤维复合材料、生物科技产业园，打造新能源、新材料、生物医药、信息技术等新兴产业集群。培育壮大以文化旅游为龙头的现代服务业，加快建设华夏文明传承创新区，推进崆峒山、大云寺·王母宫、关山三个大景区和静宁红色旅游基地建设，开发建设一批养生产业园、精品景区、旅游名镇和旅游名村，加强文化品牌传承与保护，打造全国文化旅游养生第一地；加快发展商贸物流、现代金融、科技服务、电子商务、保健养生等产业，建设陇东旱码头商贸物流中心和智慧物流集散中心。

（三）注重协调发展，促进城乡统筹和均衡发展，全力加快城乡一体化进程。

调整生产力布局，优化资源配置，促进区域之间、城乡之间、三次产业之间协调发展。以建设省域副中心城市为目标，坚持尊重自然、传承历史、集约紧凑、绿色低碳理念，推动“一中心两园区”率先发展，加快城中村、城际村和建制镇棚户区改造，实施天门塬新区、泾河北片区等区域综合开发，推进平凉工业园区、崆峒山生态文化旅游示范区产城融合，提升综合承载能力。改革完善城市规划，推进“多规合一”，加快地下管廊、城市道路、给排水、污水垃圾处理、燃气供热、绿化美化等基础设施配套，积极创建国家园林城市和省级园林县城，增强城市的宜居性。加快华亭、静宁撤县设市和泾川、崇信撤县设区进程，推进崆峒区与泾川、崇信县协同发展，连接交通、融合人文，构建百万人都市区。深化户籍和住房制度改革，加快农民工市民化进程，提高户籍人口城镇化率。推进跨区域产业联结、组团发展，支持崆峒区建设统筹城乡发展先行区，泾川、灵台两县建设国家生态文明示范县，华亭、崇信两县建设资源节约型和环境友好型示范县，庄浪、静宁两县建设现代旱作农业发展示范区，平凉工业园区创建国家级经济技术开发区。健全城乡发展一体化体制机制，推动城市基础设施和公共服务向农村延伸，建立统一的城乡居民基本养老等社会保障制度，实施改善农村人居环境行动计划，建成一批特色名镇和中心村。

（四）秉持绿色发展，加强生态建设和环境保护，全力建设国家级生态文明建设示范市。

大力实施生态文明战略，着力增加人民群众绿色福利。严格落实生态功能区保护规划，深入实施天然林保护、退耕还林等重点生态工程，推进城乡绿化、通道绿化、水系绿化和四旁绿化，加大封山禁牧力度，加强水源涵养和水源地保护，推进重点流域综合治理，构筑生态安全屏障。加快低碳循环发展，推动煤炭等化石能源清洁高效利用，鼓励绿色出行，扩大新能源汽车、电动车应用，推广绿色建筑和建材，加强重点领域节能减排，加快火电企业超低排放改造，建设一批循环经济示范基地，打造全省循环经济示范区。实行水资源消耗、建设用地总量和强度双控行动，加强雨洪资源利用、城镇低效用地再开发和工矿废弃地复垦，促进资源集约节约利用。完善污染排放许可制度，健全生态保护补偿机制，加大土壤污染治理，实施化肥、农药零增长行动，推进现有污水处理厂提标改造，实现县城、重点小城镇污水、生活垃圾全收集全处理。

（五）深化改革开放，扩大对外交流与经贸合作，全力打造“一带一路”重要节点城市。

坚持以改革促开放、以开放促开发，构建合作平台，提高对外开放水平。认真落实中央和省市委总体部署，有序推进行政管理、国资国企、金融财税、农业农村、市场体系等重点领域改革，破除体制机制障碍，释放发展活力。健全现代市场体系，建立负面清单管理制度，完善重点领域价格市场形成机制，深化公共资源交易体制改革，支持非公经济加快发展。以“一带一路”为牵引，实施互联互通、产业对接、经贸合作、人文交流、战略平台五大工程，培育提升崆峒养生文化旅游节、海峡两岸西王母论坛、皇甫谧世界针灸文化交流会、平凉金果博览会等地域性、特色性交流平台，建立多层次开放合作机制，加快向东融合、向西开放，推进与“一带一路”沿线国家和地区在资源开发、新能源、农产品加工等领域的交流合作，加强与西兰银省会城市和天水、庆阳、宝鸡、定西、固原等周边地区在商贸物流、文化旅游、交通基础设施建设等领域的分工协作，支持市内骨干企业“走出去”，积极承接东部产业转移，持续扩大招商引资，建设清真食品国际认证中心和清真食品产业园，建成平凉工业园区保税物流中心和保税仓库、公铁物流园，建设兰州新区“飞地经济”平凉产业园。

（六）坚持共建共享，提升公共服务均等化水平，全力促进民生改善和社会和谐。

坚持教育优先地位，落实立德树人根本任务，深化教育改革，促进教育公平，提高教育质量。大力发展学前教育，均衡发展义务教育，普及发展高中教育，支持甘肃医学院学科和内涵建设，建成平凉职业技术学院。新建市青少年宫、老年大学和妇女儿童活动中心，鼓励社会力量和民间资本提供多样化教育服务。实施重大人才工程，统筹推进各类人才队伍建设。鼓励支持大众创业、万众创新，建立覆盖城乡的公共就业创业服务体系，推行终身职业技能培训制度，做好公益性岗位托底安置，保障零就业家庭等就业困难人员就业，每年新增城镇就业3万人以上。实施全民参保登记计划，逐年提高城乡低保水平，积极发展职业年金、企业年金和商业养老保险，加快完善社会保险体系和救助体系。推进健康平凉建设，深化医药卫生体制改革，健全基层医疗服务体系，完善重特大疾病保障和救助机制，加快发展中医药产业，提高医疗服务水平和公共卫生防控能力。全面落实一对夫妇可生育两个孩子政策，促进人口均衡发展。推进养老服务体系建设，大力发展养老服务业，保障妇女、未成年人和老年人权益，加快发展残疾人事业。广泛开展全民健身活动，大力发展体育事业和体育产业。加强社会治理体制机制、能力和信息化建设，完善社会组织登记管理制度，规范和引导网络社团社群健康发展，推进社会治安综合治理，完善立体化防控体系，提高公共突发事件防范处置和防灾减灾救灾能力。全方位强化安全生产，全过程保障食品药品安全。强化普法教育，完善信访制度，引导公民、法人在法治框架内表达诉求、解决矛盾纠纷、维护合法权益。全面落实双拥优抚政策，加强国防教育，促进军地军民融合发展。

2016年工作任务

2016年是实施“十三五”规划的开局之年，也是全面深化改革、推进脱贫攻坚的重要一年。我们将认真贯彻中央经济工作会、省委经济工作暨扶贫开发工作会议精神，全面落实市委“十三五”规划《建议》，对接用好中央加强供给侧结构性改革所实行的宏观政策要稳、产业政策要准、微观政策要活、改革政策要实、社会政策要托底五大政策，抓好抓实去产能、去库存、去杠杆、降成本、补短板五大任务，促进全市经济社会持续健康发展，努力实现“十三五”经济社会发展的良好开局。

经济社会发展主要预期目标是：地区生产总值增长7.5%以上，规模以上工业增加值增长6%以上，固定资产投资增长15%以上，社会消费品零售总额增长10%以上，公共财政预算收入同比增长8%，城镇居民人均可支配收入增长10%以上，农民人均可支配收入增长12%以上，城镇登记失业率控制在4%以内，居民消费价格指数控制在103%以内，人口自然增长率控制在14‰以内，单位生产总值能耗和主要污染物排放完成省上控制指标。

重点抓好九个方面工作：

（一）突出精准施策，在脱贫攻坚上聚力突破。全面落实 “2+19”精准扶贫方案，推进各项政策措施到户到人，实现122个贫困村、2.48万贫困户、10.5万贫困人口脱贫，崆峒、华亭、崇信3县区脱贫摘帽。分类实施精准脱贫措施。坚持因地制宜、因村施策、因户扶持，引导有劳动能力的1.08万户4.4万人发展牛、果、菜等特色产业，输转劳务6.7万人次，实现产业脱贫；对生存条件恶劣的贫困人口，年内实施易地搬迁4507户17256人；对丧失劳动能力的3600户1.6万人进行社会救助，实现社保兜底脱贫。加强贫困乡村基础设施建设。抢抓国家加大对贫困地区支持力度的机遇，集中实施贫困村道路通畅工程，推进农村电网改造和动力电入户，提升贫困村饮水安全保障水平，加快贫困户危房改造，大力改善贫困乡村生产生活条件。强化扶贫保障。加大财政资金扶持，扩大信贷资金投入，发放专项扶贫贷款26亿元以上。统筹各类培训资源，培训贫困对象5.3万人次。完善定点扶贫机制和考核评价机制，强化驻村干部管理，靠实帮扶单位责任，推动双联行动与精准脱贫深度融合。

（二）突出项目支撑，在扩大固定资产投资上聚力突破。适应新常态下国家推进结构性改革、扩大有效供给的政策导向，深入实施 “3341”项目工程，精心谋划和实施一批能够加快转型、夯实基础、改善民生的重大项目，以项目拉动投资、支撑发展。加快推进项目前期。紧扣 “十三五”规划，深入论证，积极对接，争取陇东特高压电力外送工程列入国家“十三五”能源和电力发展规划，完成泾川盘口、华亭后河、庄浪梁河水库等骨干水源工程前期，推动宝中二线、西平二线、定西至平凉至庆阳高铁、平凉城市轨道交通、灵台至华亭高速、白龙江引水支线工程、静宁330千伏变电站项目前期工作取得实质性进展。提升招商引资层次。突出招大引强，开展一对一、点对点精准招商，加强光伏铸锭、锂离子电池、数字文化产业园等意向性项目的洽谈引进，加快红星美凯龙城市综合体、万达广场商业综合体、智慧城市等已签约项目落地进度，实施招商引资项目300项以上，到位资金增长12%。推进重大基础设施建设。加快实施“6873”交通突破行动和“6363”水利保障行动，开工建设平庆高速和平泾一级公路，加快平天、彭大高速、国道312线平凉西至界石铺、平华一级公路建设进度，建成平凉客运枢纽站和快速客运站主体工程，改扩建平凉汽车西站，力争平凉机场开工；开建崆峒水库改扩建工程，加快引洮二期静宁受水区配套工程建设，实施灵台新集水库建设和华亭王峡口水库改扩建等水利工程65项；加快尚家塬330千伏变电站扩建，新建改建华亭杨庄、庄浪水洛、平凉柳湖110千伏变电站，持续推进农网改造。加快3G、4G移动通信和高速无线移动互联网建设，实施“宽带乡村”示范工程，建设平凉云中心和地理信息系统，推进光网城市建设。

（三）突出提质增效，在特色农业开发上聚力突破。以提高农业质量效益和竞争力为核心，推动农业产业优化、产出高效、产品安全，促进农业增效、农民增收。着力优化农业产业布局。实施现代农业“五个百万”提质增效工程，推广旱作农业技术229万亩，粮食产量稳定在110万吨以上。突出肉牛科学养殖、全产业链开发，新建标准化养殖小区30个，牛饲养量保持在116万头以上。加快果产业品种更新改优，推广矮化密植栽培，创建果品标准化生产、绿色有机和全球良好农业示范基地150万亩，果品总产量达到180万吨。种植脱毒种薯100万亩。启动建设泾汭河川区国家级绿色蔬菜产业园，新增设施蔬菜2万亩，蔬菜总产量达到150万吨。大力发展木本油料，新植油用牡丹5万亩。支持发展中药材、小杂粮等区域性特色产业。着力提升农业生产水平。突出节水强农，新增有效灌溉面积6000亩，发展节水灌溉面积3万亩，建设高标准农田8.5万亩。提高农业机械化装备水平，增强农业综合生产能力。建成海升智能温室工业化栽培生态示范项目。健全完善农产品质量安全可追溯体系，推进农业标准化，认证无公害农产品产地、绿色食品原料基地10万亩。着力壮大新型农业经营主体。推进土地规范有序流转，鼓励发展适度规模经营，加快培育新型职业农民，发展一批家庭农场，创建农民专业合作社200家，新建改扩建龙头企业25户，形成“公司+基地+农户”产业经营体系，让农户更多分享农业产业链增值收益。

（四）突出优化升级，在培育工业经济增长点上聚力突破。坚持传统产业改造提升与新兴产业培育壮大并重，着力去产能、去库存、降成本，促进工业经济企稳回升。推进平凉千亿级煤电化冶产业链建设。加快邵寨、赤城、五举等矿井建设进度，力争唐家河煤矿获得国家核准，安家庄煤矿取得国家发改委“路条”，开工建设华亭电厂二期、70万吨烯烃、660万吨煤基新燃料和6万吨镁合金深加工循环经济生产线，加快20万吨聚丙烯项目建设。促进传统产业改造提升。启动虹光、红峰出城入园改造，实施平凉工业园区供电侧改革试点，完成地方煤矿企业兼并重组，推进困难企业扭亏脱困，开工建设石油、矿山机械高端装备制造项目，建成兰煤矿山机械再制造基地、5000辆新能源汽车改装生产线和庆华陶瓷、鑫盛建材二期。加快天纤棉业20万锭棉纱、昊源医药物流园等项目建设。培育壮大战略性新兴产业。加快平凉光伏产业园建设，推进泾川、庄浪、静宁集中式光伏发电和静宁、庄浪风力发电项目，庄浪中药饮片、海洋浮力补偿系统材料项目年内建成投产。推进工业园区（集中区）建设。以创建国家级经济技术开发区为目标，进一步创新平凉工业园区管理运行机制，配套完善基础设施，建设中小企业创业园，年内新入园亿元以上项目6个，千万元以上项目15个，固定资产投资增长20%，规模以上工业增加值增长11%。加快平凉工业园区、华亭工业园区循环化改造，组建华亭、静宁、泾川、庄浪工业园区（集中区）投融资企业。

（五）突出融合互补，在扩张三产总量上聚力突破。探索文化旅游融合发展新模式，加快商贸物流体系建设，大力发展现代服务业，三产增加值增长10%以上。加快大景区建设。推进崆峒山旅游配套设施二期等重点项目建设，加快崆峒山、大云寺·王母宫管理体制改革，启动关山大景区规划建设，成立大景区管理委员会，组建旅游集团公司并投入运营。大力发展乡村旅游、游园采摘、民俗体验、滑雪漂流，加快融入西兰银黄金旅游圈，串联包装精品线路，创新宣传营销方式，旅游接待人数、综合收入分别增长20%和22%以上。推进中华崆峒养生地建设。开建平凉养生商贸城，建设崆峒道文化、大云寺禅修等七大养生园，加快培育骨干文化企业和养生产业，扩大文化企业、养生服务项目入园，完成文化产业投资20亿元以上，文化产业增加值增长15%。繁荣壮大商贸物流业。加快义乌国际商贸城等重点商贸设施建设，培育发展信息快递、仓储配送等配套产业，推进商贸物流业集约集群发展。积极发展融资租赁、家政服务等生产生活性服务业。加快电子商务示范县、示范基地和示范企业建设，建成泾川、华亭、灵台3个县级农村电子商务中心。

（六）突出中心带动，在新型城镇化发展上聚力突破。健全完善规划体系。加快省列新型城镇化试点县镇 “多规合一”规划报批实施，完成16项县城专项规划编制和8个乡镇总体规划、19个村庄建设规划修编，启动22个美丽乡村规划编制，实现县城重要专项规划全覆盖。推进中心城市建设攻坚提质。坚持点上突破、线上延伸、面上提质，实施火车站、东大门、绿地公园、盘旋路等6处节点风貌塑造工程，打造城市标志性建筑；实施泾河大道东段、东环路拓建等14条城市道路建设项目，完善城区路网；加强综合管廊、垃圾转运站、便民市场等公共设施建设，增强城市综合服务功能；用足用活国家政策，实施天门塬、双桥路、太统路等12个片区棚户区改造，加大货币化安置，增强城市可持续发展能力。加快县城、小城镇和新农村建设。抓好省列“两县三镇”和市列八镇新型城镇化试点，加快县城、重点小城镇供水、供气、集中供热、污水垃圾收集处理等基础设施配套，提升综合承载能力。抓建省级美丽乡村示范村20个以上、环境整洁村200个，建成市县级美丽乡村示范村40个。提升城市管理水平。进一步理顺城市管理体制，推进环境卫生、市容市貌、交通秩序、市政设施智能化、网格化、精细化管理，加大综合执法和拆违治乱力度，推行环卫作业市场化运行模式，着力解决脏乱差问题。加快户籍制度改革，推进农民工市民化。打通安置房和商品房、保障性住房对接通道，多措并举消化库存商品房，促进房地产市场稳定发展。

（七）突出绿色发展，在生态文明建设上聚力突破。加强生态建设与保护。实施天保二期、三北五期、第二轮退耕还林、重点公益林补偿等生态工程，推进流域治理、梯田建设和土地整理，开展大规模造林绿化行动，完成城乡造林20万亩，新修梯田18万亩，治理水土流失300平方公里，治理河堤30公里。强化污染减排。加大大气、水、土壤等污染防治和环境综合整治力度，严格高耗能、高污染项目审批管理。实施一批污染减排项目，加强重点行业、重点企业环境执法监管。全面推行排污许可管理，严控污染物排放。促进资源集约节约利用。严守耕地红线，加大园区项目投资强度，提高城镇土地利用效率。落实最严格的水资源管理制度，加强节水型企业建设，提高污水再生利用率和中水回用率。加强公共机构节能管理，实施节能、循环经济及资源综合利用重点项目20项，推进企业循环化生产、产业循环化组合、园区循环化改造。

（八）突出普惠共享，在民生工程建设上聚力突破。优化资源配置，落实公共普惠政策，集中实施10项为民办实事工程，全市财政用于民生的投入比重达到80%以上。1.政务服务便民工程。开建市政务服务中心综合楼；新建改扩建崆峒、泾川、庄浪3县区政务服务中心；建成60个标准化乡镇政务服务中心、300个多功能村级便民服务站；整合各部门便民热线资源，重组建设市综合便民服务热线系统平台，构建“12345”一号受理、互通联动、统一监管、高效便捷的办理体系和运行机制。2.社会保障惠民工程。落实优惠政策，提供就业岗位3万个；城乡低保标准提高10%；提高城镇基本医疗保险财政补助标准和报销比例，有序扩大异地结算范围；开建市老年公寓。3.安居惠民工程。实施棚户区改造8311户；新建公租房2636套；改造农村危房3万户。4.饮水安全工程。开建韩家沟水厂，提高中心城区供水保障能力；新建精准扶贫饮水安全工程22处，解决3.04万户13.9万人饮水不稳定问题；加强供水安全监管，供水保证率、水质达标率达到90%以上。5.教育惠民工程。新建改建义务教育学校校舍8万平方米；改建村级幼儿园385所；建成标准化学校222所；建成平凉职业技术学院并挂牌招生。6.卫生保障工程。建成市级儿童专科病区；新建市级中医医院（皇甫谧针灸医院）；改扩建静宁、庄浪、华亭县中医院和泾川县妇幼保健院；建成16个乡镇卫生院附属工程和5个乡镇卫生院职工周转宿舍；新建标准化村卫生室50个。7.市政便民工程。建设新民路十字人行天桥和盘旋路南侧、柳湖公园与绿地公园之间地下通道；建设一批中心城区、县城公共停车场，划定停车区域，解决停车难问题。8.文化体育惠民工程。建成市博物馆主体；开建市体育中心；建成20个乡镇综合文化站、294个贫困村文化服务中心（乡村舞台）；实施中央和省市县四级广电节目无线数字化覆盖、广电网络乡村通工程；建设乡镇体育中心8个、行政村农民健身工程120个；启动市科技馆、青少年宫前期工作。9.道路通畅工程。实施建制村通畅工程738公里，实现100%的建制村通沥青（水泥）路；完成5600公里通村硬化道路安全隐患治理。10.法律服务惠民工程。建立法律服务网络平台和法律援助对象动态数据库，实现“法律服务直通车”全覆盖，办理法律援助案件3500件以上。

与此同时，全面推进各项社会事业协调发展。加快构建立体化社会治安防控体系，健全重大事项社会稳定风险评估、化解矛盾纠纷综合调处和维稳应急处置机制，推行涉法涉诉信访依法终结制度。依法严厉打击各类违法犯罪活动，有效预防和减少不安全不稳定因素。严守安全生产红线，强化打非治违和隐患排查治理，严格目标管理与责任追究，杜绝重特大生产安全事故。健全基层食品药品监管体系，加大专项整治力度，保障人民群众饮食用药安全。

（九）突出改革创新，在破解发展难题上聚力突破。着力增强改革意识，提高改革能力，推动重点领域改革措施落地见效。深化行政审批制度改革。持续推进简政放权，放管结合，优化服务，建立行政审批中介服务收费目录管理制度。进一步完善“三张清单一张网”，加快实施“网上行权”。继续推进商事制度改革，加快建设企业信用共享体系，加强事中事后监管。实行政府定价项目清单化管理。深化公共资源交易体制机制改革。加快不动产统一登记。完善政府购买公共服务制度，扩大购买范围。培育壮大市场主体。落实支持非公经济发展的各项政策措施，优化劳动力、土地、资金、技术、管理等要素配置，大力推动大众创业、万众创新，激发非公有制经济活力和创造力，非公经济增加值增长11%。加快推行PPP等融资模式，吸引带动更多社会资本参与项目建设。深化农村改革。全面完成农村土地承包经营权和果树经济林确权登记颁证，加快建设县级农村土地承包经营权流转交易平台和仲裁机构，推进林业综合改革试验示范区建设和国有林场改革。加快水权、水价、水利建管体制改革。深化供销合作社综合改革。抓好市级政策性农业保险扩面提标。启动农房保险试点。推进财税金融改革。健全政府预算体系，实施财政中期规划，推行全口径财政预算，建立事权与支出责任相匹配的财税制度。推动兰州银行平凉分行开业运营，争取省股权交易中心在平凉工业园区设立运营中心。推进普惠金融，创新农村金融服务，提高金融服务均等化水平和金融支持精准脱贫的有效性。规范发展互联网金融新业态，加大上市企业、股权投资类企业培育和政策扶持，力争在“新三板”挂牌上市企业2户以上。加强金融机构监管，确保金融市场稳定。统筹推进科技体制、公务用车等方面改革。

加强政府自身建设

适应新常态，谋求新发展，对政府自身建设提出了更高要求。我们将勤勉尽责，主动作为，更好地担当起推进脱贫攻坚、实现全面小康的历史重任，对人民负责、受人民监督、让人民满意。

严格依法行政。进一步强化法治思维，弘扬法治精神，增强全社会特别是公务人员尊法学法守法用法观念，不断提高依法行政的能力和水平。坚决执行人大及其常委会的决议、决定，自觉接受人大法律监督和政协民主监督，认真办理人大代表建议和政协委员提案，支持各民主党派开展工作。坚持法定职责必须为、法无授权不可为，进一步完善落实公众参与、专家论证、风险评估、合法性审查、集体讨论决定等决策制度，不断提高行政决策的科学化、民主化、法治化水平。加大综合执法力度，规范行政许可、行政处罚、行政强制、行政检查等程序，建立执法全过程记录制度，从根本上解决多头执法、多头检查和乱收费、乱罚款等问题。

持续改进作风。巩固和扩大党的群众路线教育实践活动、“三严三实”专题教育成果，严格执行中央八项规定和省市委改进作风相关规定，驰而不息纠正“四风”。深化机关效能建设，坚决纠正不作为、慢作为、乱作为等懒政怠政行为，严肃查处对待群众态度蛮横、作风粗暴等影响政府形象的人和事。深入推进电子政务建设，扩大行政权力运行、公共资源交易等领域信息公开，完善政府新闻发布制度，及时回应社会关切，提高政府工作透明度。

提高执行能力。严明执行责任，加强履责监管，强化督查考核，保障重大决策、重大项目、重点工作落实。重视加强对国家重大政策、全市发展重大问题的研究，增强指导推动工作的针对性、实效性。建立健全正向激励和整改问责长效机制，为想干事、能干事的干部提供更大舞台，形成竞相发力、破难前行的良好氛围。强化效能监察，坚决查处执行不力、失职渎职、弄虚作假等行为，确保政令畅通。

加强廉政建设。坚持把纪律和规矩挺在前面，认真贯彻《中国共产党廉洁自律准则》和《中国共产党纪律处分条例》，严格落实党风廉政建设主体责任和监督责任，强化政府内部权力监督制约，在权力集中部门和重点岗位探索推行分事行权、分岗设权、分级授权。强化政府投资项目监管，严控工程建设规模标准，坚决杜绝重复建设和超预算、超标准建设。加强审计监督，严肃查处吃拿卡要、暗箱操作、权力寻租、贪污腐败等违法违纪行为，努力做到干部清正、政府清廉。

各位代表，新的目标催人奋进，新的征程任重道远。让我们更加紧密地团结在以习近平同志为总书记的党中央周围，在省委省政府和市委的坚强领导下，凝心聚力、奋发有为，开拓进取、扎实工作，为与全国全省一道全面建成小康社会而努力奋斗！
